# Locus Coeruleus in Non-Mammalian Vertebrates

**DOI:** 10.3390/brainsci12020134

**Published:** 2022-01-20

**Authors:** Sijia Wang, Zhirong Wang, Yu Mu

**Affiliations:** Institute of Neuroscience, State Key Laboratory of Neuroscience, Center for Excellence in Brain Science and Intelligence Technology, Chinese Academy of Sciences, 320 Yue-Yang Road, Shanghai 200031, China; sjwang@ion.ac.cn (S.W.); wangzr@ion.ac.cn (Z.W.)

**Keywords:** locus coeruleus, norepinephrine, vertebrates, non-mammals, fish, amphibian, reptile, bird, anatomy, function

## Abstract

The locus coeruleus (LC) is a vertebrate-specific nucleus and the primary source of norepinephrine (NE) in the brain. This nucleus has conserved properties across species: highly homogeneous cell types, a small number of cells but extensive axonal projections, and potent influence on brain states. Comparative studies on LC benefit greatly from its homogeneity in cell types and modularity in projection patterns, and thoroughly understanding the LC-NE system could shed new light on the organization principles of other more complex modulatory systems. Although studies on LC are mainly focused on mammals, many of the fundamental properties and functions of LC are readily observable in other vertebrate models and could inform mammalian studies. Here, we summarize anatomical and functional studies of LC in non-mammalian vertebrate classes, fish, amphibians, reptiles, and birds, on topics including axonal projections, gene expressions, homeostatic control, and modulation of sensorimotor transformation. Thus, this review complements mammalian studies on the role of LC in the brain.

## 1. Introduction

The locus coeruleus (LC) is a vertebrate-specific norepinephrinergic (NE) nucleus [1,2,3,4]. The LC locates deeply in the dorsal part of the brainstem [5], where a small number of neurons extensively branch their axons and provide the main source of NE to the brain [6]. Intriguingly, along the evolutionary path, animals develop more LC cells, probably due to demand for commanding more brain nuclei and influencing more complex physiological and behavioral responses. For example, as a member of the most ancient group of vertebrates, zebrafish LC has only 10–20 NE cells [7,8]. Songbirds have ~700 and quails have ~1300 LC cells [9]. Mammals possess more LC neurons than birds, rats have 3000 LC cells, and monkeys have 7000 LC cells [10]. The number of LC neurons correlates with the overall number of neurons across non-mammalian species. As one might expect, humans have the most LC cells, up to 50,000, to cope with arguably the most sophisticated brains [11].

Comparative studies combine observations from different model organisms. Among vertebrate species, remarkable conservation at multiple scales has been reported in LC, including a relatively homogeneous cell identity [12], broad axonal projections [13], and general roles in enhancing arousal and alertness [14]. For example, the whole-brain screening in larval zebrafish guided the discovery in rodents that multiple parallel neuromodulatory systems, including the LC, coordinately modulate brain states [15]. Among vertebrates, the shared traits of LC across the molecular, synaptic, circuit, and system levels make this highly complex brain nucleus an ideal system for comparative analysis.

Here, we review the literature from non-mammalian model organisms (fish, amphibians, reptiles, and birds) on LC regarding anatomical projections, gene expression profile, and sensorimotor transformation, and compare them with the progress in mammalian studies to summarize current understanding and inspire new directions of LC research.

## 2. Axonal Projections of the LC

**Figure 1:**
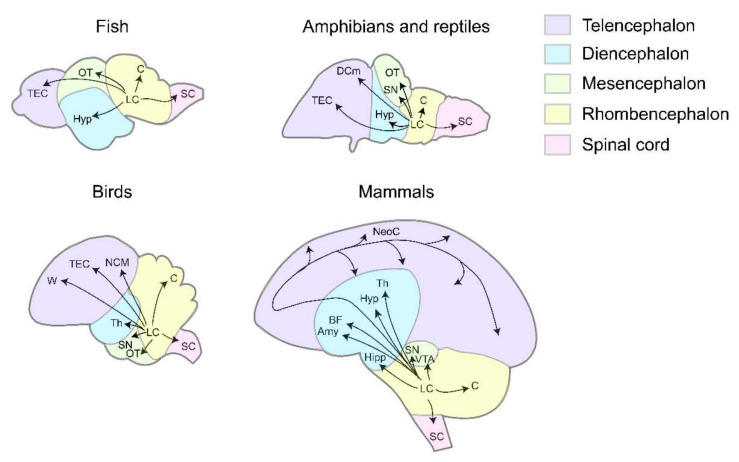
The axonal projection patterns of LC in different vertebrates. In fish, LC axons project to TEC, OT, Hyp, C, SC [13,18,19,20]. In amphibians and reptiles, LC axons project to TEC, DCm, Hyp, SN, OT, C, SC [21,22,23,24,25,26,27,28,29,30]. In birds, LC axons project to TEC, W, NCM, Th, SN, OT, C, SC [31,32,33,34,35,36,37,38]. In mammals, LC axons project to NeoC, BF, Hyp, T, Amy, Hipp, SN, VTA, C, SC [12,39,40,41,42,43,44,45,46,47]. Puple, telencephalon. Blue, diencephalon. Green, mesencephalon. Yellow, rhombencephalon. Pink, spinal cord. TEC, telencephalon. DCm, dorsal medial cortex. W, wulst. NCM, caudomedial nidopallium. NeoC, neocortex. Hyp, hypothalamus. Th, thalamus. BF, basal forebrain. Amy, amygdala. Hipp, hippocampus. OT, optic tectum. SN, substantia nigra. VTA, ventral tegmental area. C, cerebellum. SC, spinal cord.

Extensive axonal projections of the LC enable neural modulation at the whole-brain scale. In both mammals and non-mammalian vertebrates, conservations of anatomical features can be observed at multiple levels, such as LC axons bearing varicosities [13,16,17]. Most importantly, at the circuit level, axonal projections from the LC form the conserved ascending pathways-reaching the telencephalon (TEC), diencephalon (DEC), mesencephalon (MEC), and the conserved descending pathways-reaching rhombencephalon (REC) and the spinal cord (SC) (Figure 1).

### 2.1. Projection Targets

#### 2.1.1. Telencephalon

In mammals, the LC broadly innervates multiple regions in the telencephalon to mediate attention, arousal, and sensory processing [12,48,49,50]. Similar projection patterns exist in non-mammalian vertebrates. In fish *Polypterus palmas*, telencephalon receives bilateral LC projections [18]. Within the telencephalon of zebrafish *Danio rerio*, the olfactory bulb, subpallium, striatum, midline septum, and anterior commissure are targeted [19,20]. In newt *Triturus cristatus*, lizard *Varanus exanthematicus*, and frog *Rana perezi*, LC fibers extend from the isthmic segment to the striatum and septum [21,22,23]. Within the telencephalon of the pigeon *Columba livia*, projection targets of the striatum can be further divided into the paleostriatum augmentum, neostriatum, archistriatum, and hyperstriatum [31]. Striatum and septum in telencephalon are the common targets of the LC among vertebrates, whereas the projections to the cortex evolve as new ascending pathways to the telencephalon. The LC neurons project to the dorsal medial cortex (DCm) in lizard *Gekko gecko* [24], and the secondary auditory cortex (the caudomedial nidopallium, NCM) and the wulst in zebra finch *Taeniopygia guttata* and pigeon *Columba livia* [33,34], indicating their involvement in species-specific functions such as generating bird songs.

#### 2.1.2. Diencephalon

The common targets of LC axons in the diencephalon are the thalamus and the hypothalamus in zebrafish *Danio rerio*, lizard *Varanus exanthematicus*, and pigeon *Columba livia* [20,25,32]. In pigeons, the LC projects to the limbic system and striatal complex, overlapping more with dopaminergic efferents than in mammals [32]. Moreover, all the major NE receptors are present in the amygdala and the hippocampus, confirming the impact of the LC-NE system on emotions, learning, and memory [41,42,43].

#### 2.1.3. Mesencephalon

Optic tectum (OT) proves to be the biggest target region of LC NE projections in the mesencephalon of zebrafish *Danio rerio*, frog *Rana perezi*, frog *Xenopus laeuis*, and pigeon *Columba livia* [13,20,27,28,36]. These projections form pathways that contribute to the visual alertness in zebrafish and the dark/light adaptation in amphibians [15,27,28]. The arborization field of LC neurons in the OT expanded during the course of evolution. In fish, the LC only spreads a few collaterals extending caudally and ventrally to the OT [13]. In amphibians, the LC projects predominantly to the superficial, recipient tectal layers [28]. In birds, the LC projection arborizes densely in layers 2, 4, and 7, suggesting a potent modulation of visual processing [36]. Apart from the OT, the substantia nigra (SN) in frog *Xenopus laevis* and chicken *Gallus domesticus* receives LC projections [26,35]. A similar pathway from the ventral tegmental area (VTA)-DA system to the LC-NE system exists in mammals, supposedly conveying the reward prediction signal from the dopaminergic system to the NE system [51]. However, such DA to NE pathway is not identified in fish. Therefore, it might have appeared later in the course of vertebrate evolution.

#### 2.1.4. Rhombencephalon

In the descending pathways, projections from the LC first enter the cerebellum. In zebrafish *Danio rerio* and turtle *Pseudemys scripta elegans*, LC axons enter the cerebellum, with little path or region specificity [13,20,29]. In chicken *Gallus domesticus*, however, the LC forms region-specific projections to the cerebellum: the caudal LC forms a latero-caudal bundle in the inferior cerebellar peduncle, while the rostral LC forms a medio-rostral bundle in the superior peduncle [35,38].

#### 2.1.5. Spinal Cord

In all the vertebrates, the LC substantially projects to the spinal cord [20,30,37,45,46,47]. In mammals, LC projections inhibit sensory neurons in the dorsal horn [52,53] and activate motor neurons in the ventral horn by acting on different types of NE receptors [2,39,44,54]. The LC also projects to sympathetic and parasympathetic preganglionic neurons located in the intermediolateral cell column (IML) of the spinal cord and exerts intricate effects involving both excitatory and inhibitory depending on the NE receptor types [55,56,57]. In fish, the TH-reactive fibers are observed to enter the spinal cord, predominantly through the ventrolateral pathway [58]. While there is little evidence on the impact of LC in any specific motor behaviors, those TH+ fibers may form a close connection with motor neurons in the spinal cord and influence any motor-related behaviors.

### 2.2. Future Directions for Mapping LC Wiring Diagram

The above studies show conserved projections of the LC among vertebrates. However, these projections have been mapped at the populational level by anterograde/retrograde dye tracing [32,37,41,44,45,59,60,61], or immunostaining [13,62,63,64]. Next, a single-cell level wiring diagram will be important. For example, although neuronal ensembles fire asynchronously in the LC [65], it is unknown if this heterogeneity is maintained in downstream areas by “specific” projections (groups of LC neurons preferentially target different brain regions), or erased by “uniform” projections (different LC neurons randomly project to brain regions) [12,66,67,68]. Two promising technologies include sparse labeling and high-throughput sequencing. Examples are the MouseLight project carried out by Janelia Research Campus, reconstructing ~1000 projection neurons in the mouse brain [69], and the Multiplexed Analysis of Projections by Sequencing (MAPseq) developed by Anthony Zador’s lab at the Cold Spring Harbor Laboratory [68].

Neither the MouseLight project nor the MAPseq project can completely reconstruct the LC wiring at single-cell resolution due to low throughput or lost spatial details. However, studies in larval zebrafish may provide the first complete dataset for LC wiring. In larval zebrafish, within a short time period, hundreds of LC neurons can be reconstructed and mapped onto a common brain [70,71,72]. Because the number of neurons in larval zebrafish is two orders of magnitude less than that in mice, such datasets will oversample the LC and lay a solid foundation for further characterization. The projection profile of a single LC neuron will be covered as well. Next, with mature technology on brain-wide functional imaging, neuronal activities, and NE release can be integrated into this projection database [73,74,75,76]. Together, a complete LC functional projectome will substantially advance the understanding of LC systems in vertebrates.

## 3. LC Development

These complex projections are established from the morphogenesis of the LC during early development. In birds, the formation of the LC generally takes three steps-starting with loose neuroblasts, followed by the distinction between the dorsal part and ventral part during the incubation stages, and finally are equipped with rich and specific projections at the age of 6 weeks [5]. The branching pattern of dendrites and the volumetric alterations of individual LC neurons continue to increase during the last days of embryonic development and after hatching [5]. Similarly, in fish, amphibians, and birds, all the neuronal populations and most of the projections are detected by the end of the embryonic period [7,77,78,79,80]. Notably, the LC cells in embryos are always observed in positions corresponding to their adult location, suggesting that they do not migrate much during development [81].

## 4. Unique Gene Expression in LC

A unique gene expression profile gradually unfolds in LC. In mammalian LC, more than 3000 genes are highly expressed and more than 100 genes show significant sex differences [82,83]. In zebrafish, a mutant line called *soulless* completely loses LC NE neurons, leading to the discovery of the master control gene for generating these neurons. The homeodomain protein Phox2a integrates the signal from morphogens such as bone morphogenic protein (BMP) and fibroblast growth factor-8 (FGF8), in turn, triggers the expression of NE synthesis enzymes, tyrosine hydroxylase (TH) and dopamine β-hydroxylase (DBH), and generates functional LC [84].

The soma size of LC NE neurons is generally larger than the neurons in the immediate vicinity [17,58,81,85]. This phenomenon could be related to a high expression level of nerve growth factor (NGF) receptors in the LC [86]. In chicken, LC neurons express a high level of neurotrophin receptors TrkA and p75, and in the presence of neurotrophin NGF, these neurons grow bigger and are rescued from the toxic effect of 6-hydroxydopamine [86]. Interestingly, two parallel routes were identified for trafficking the NGF from the richest source (such as the retina) to LC soma. One pathway is transporting NGF by axon tracts, anterogradely by other neurons (such as retinal ganglion cells), and retrogradely by LC axons. The second pathway is through a specialized glial/ependymal cell type, the tanycyte, which extracts NGF from the cerebrospinal fluid (CSF) and delivers it to the LC [87]. However, in mammals, the LC NE neurons do not transport NGF and lack neurotrophin receptors. Therefore, they do not respond to NGF [88]. Such differences in gene expression may cause divergence of projection patterns and circuit functions between species.

Unique gene expression profile gives rise to distinct cellular and circuit functions. Another example comes from metal transporters. The LC contains the highest concentration of copper than any other brain regions [89]. On the one hand, copper is required for metalating DBH to convert DA into NE [90]. On the other hand, copper could trigger cellular stress from its high redox activity and is associated with Parkinson’s disease and other neurodegenerative diseases [91,92].

A recent study in zebrafish led to a more thorough and surprising finding of the physiological relevance of copper to the LC activity [93]. Copper Fluor-4, a synthesized copper sensor, was developed and used to detect the copper level in vivo. Using this tool, Xiao et. al. found that copper distribution was heterogeneous in the larval zebrafish brain, and a high level of copper in LC was critical for rest-activity balance and arousal responses. The concentrated copper depends on a protein highly and specifically expressed in LC-ATP7A, a copper transporter [93,94]. Phylogenetic analysis showed that the gene atp7a comes from gene duplication in early chordates. Interestingly, the NE transporter, NET, also appears first in Gnathostomata. Such parallel evolution provides evidence that the LC-NE system was restricted in vertebrates. In Parkinson’s disease patients, decreased copper levels were found in both the substantia nigra and the LC [95], and in Wilson’s disease, mutations in the copper transporter ATP7B may lead to copper accumulation in patients [96]. In both diseases, copper-related treatments such as a low-copper diet or copper supplementation are proposed as therapeutic strategies [36,97]. Due to the specificity of copper transporter expression and concentrated copper in the LC, tools monitoring copper or copper-related biomarkers will be useful for measuring LC functions and diagnosing neurodegenerative diseases.

## 5. LC Regulates Body Homeostasis

Through the extensive axonal projection network, the LC-NE system modulates a broad spectrum of brain functions in mammals and non-mammalian vertebrates alike. For instance, the LC mediates transitions of brain states between sleep/anesthesia and wake/alertness to sustain internal homeostasis. In larval zebrafish, the normal activity of the LC maintains the awake state and promotes the sleep to wake switch triggered by sensory stimuli [98]. A similar effect was observed during general anesthesia. General anesthetic drugs decrease LC neural activity by reducing both its intrinsic excitability and synaptic inputs. Impairing LC delays the recovery from the anesthetic state [99].

The LC NE neurons are directly thermosensitive. In slice recordings from amphibian (frog *Lithobates catesbeianus*) LC neurons, when the temperature fluctuates within a naturalistic range (from 10 °C to 30 °C), the basal firing rate decreases during warming and increases during cooling [100,101]. Hyperpolarization-activated current (*I*_h_) is proposed to mediate cooling-induced firing rate increase in amphibians [101]. In mammals, though unclear in LC, neuronal firing rate from the slice recording of other brain regions, such as dorsal raphe and hypothalamus, is positively correlated with temperature fluctuation [102,103]. Moreover, although deep brain temperature is much less affected by environmental changes, pyrogen-induced fever or ambient heating could nonetheless increase the firing rate of the LC NE neurons in cats [104]. To address the difference between endotherms and ectotherms in brain thermo-environment, it would be informative to test if LC neurons in mammals are also thermosensitive and if the same *I*_h_ current responds to temperature.

Furthermore, the LC NE neurons are chemosensitive [105,106]. In amphibians, reptiles, and mammals, the firing rates of LC neurons increase in more acidic environments [100,105,107,108,109]. In response, enhanced LC activity promotes respiration [105,110], which reduces the CO_2_ concentration and increases the body temperature [111,112,113]. Unlike in the endotherms, the CO_2_/pH in ectotherms fluctuates with body temperature. An intriguing phenomenon is that acid-base homeostasis is maintained even when the CO_2_/pH changes with temperature [114]. This control process is potentially mediated through a compensatory mechanism involving the LC. In amphibians, the chemosensitivity of LC is not constant but rather positively correlates with temperature [100], allowing for adaptive respiration regulation that better responds to the fluctuation of body temperature.

Further investigations should answer two key questions. First, from ectotherms to endotherms, how have these homeostatic regulation functions been preserved, lost, or transformed. Second, could the LC neurons receive environmental signals through either local sensors in LC neurons [102] or afferent connections carrying information from sensory organs [115]. Distinguishing and combining these two input sources would advance the understanding of LC NE function in homeostatic control.

**Figure 2:**
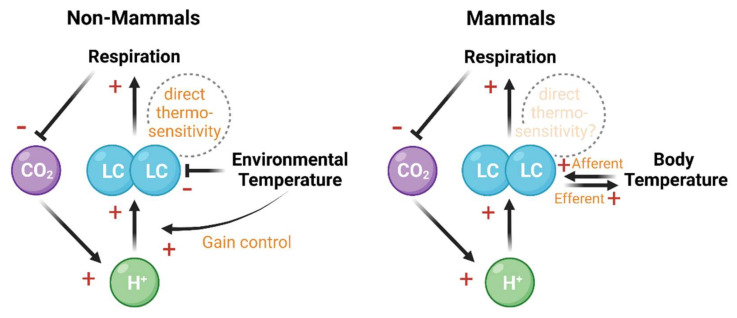
The LC integrates and regulates the chemosensitive and thermosensitive information flow in ectothermic non-mammals (**left** panel) and mammals (**right** panel). In both non-mammals and mammals, the decrease of pH enhances LC firing rate [108,109], which in turn promotes respiration and reduces retaining CO_2_ [111]. Apart from this chemosensitivity-based negative feedback, the thermosensitivity of LC forms a regulatory pathway with respiration and temperature changes. In ectothermic non-mammals, the environmental temperature negatively regulates the basal firing rate of LC, but positively modulates the chemosensitivity of LC [100,101,105,107,120]. In mammals, body temperature change and LC activity reciprocally interact with each other [104,112,113,121,122]. Direct thermosensitivity of LC has been shown in non-mammals [100] but remains elusive in mammals. Created with BioRender.com.

Neural function, body temperature, and bodily fluid acidity depend on each other and together constitute a system that dynamically adapts to the environment [100,115,116,117,118,119]. The LC NE neurons integrate multiple sources of homeostatic signals and provide feedback control through homeostatic mechanisms such as respiration (Figure 2). The above mechanisms allude to a broader role of LC in evaluating environmental feedback to optimize behavioral strategies and preserve body homeostasis. 

## 6. LC Modulates Sensorimotor Transformation

During sensorimotor transformation, three major components-LC activity, sensory processing, and motor generation interact with each other and form a dynamic system. Behavioral paradigms involving continuous sensorimotor streams, such as songbird singing or zebrafish swimming, are especially suitable for delineating these interactions. In these paradigms, LC-NE signals modulate multiple sensorimotor areas to produce flexible and adaptive behaviors.

Songbirds recognize and prefer conspecific songs for mating and vocal tutoring. The song generation system in songbirds receives wide LC projections [9], which provide NE signals that modulate both sensory processing and motor generation. In the sensory processing pathway, the LC modulates the activity of the caudomedial nidopallium(NCM), a region analogous to the mammalian secondary auditory cortex. Upregulating the NE signal in NCM enhances signal detection by reducing spontaneous firing rates and increasing the signal-to-noise ratio [123]. On the other hand, downregulating the NE signal by inducing lesion at LC disrupted the selectivity between the bird’s own song, conspecific songs, and heterospecific songs [124]. In the motor generation pathway, the NE signal modulates the singing states. Increasing NE signal in the nucleus of the arcopallium (RA), a motor nucleus critical for song generation, promotes the production of a robust song (“exploitation state”, in the presence of females), whereas decreasing NE enhances the production of variable songs (“exploration state”, singing alone) [125]. Similarly, directly stimulating the LC neurons promoted “exploitation state” [125], potentially by modulating the song variability pathway: basal ganglia-anterior nidopallium (LMAN)-RA [126,127]. In this pathway, the NE signal suppresses the firing of spiny neurons in the basal ganglia and the input from LMAN to RA [127,128].

These studies demonstrate the influence of LC-NE on sensory and motor circuitry. The system is further confounded by motor feedbacks on sensory input. Novel environment or unexpected sensory feedback triggers bursting activity in the LC [129]. Conversely, the LC modulates sensory processing, reflected by enhanced alertness or arousal and altered synaptic plasticity [15]. A similar reciprocal influence exists between the LC and motor activity. A theoretical framework was established in the last decade, proposing that the phasic activity of LC is an interrupt signal that represents “unexpected uncertainty”, and, in turn, modulates sensorimotor transformation [130,131,132]. This process may be the foundation of various forms of adaptive behaviors. However, such as the “three-body problem”, interactions that are easy to describe between two subjects could appear chaotic when three or more subjects are involved. Simultaneously recording from, and perturbing the sensory, motor, and the LC systems could enable quantitative modeling that would greatly enhance our understandings.

A study on swimming generation in zebrafish considered the three components in the same context. Larval zebrafish are placed in a virtual reality environment in which visual feedbacks reflect intentions to swim [75]. Appropriate feedback encourages zebrafish to continue swimming. The system allows for fast and repeated adjustments of feedback strength. Alternatively, there could be no feedback at all, in which case the swimming attempts would reduce or cease. During this sensorimotor loop, motor outputs and sensory feedback are constantly evaluated to generate “expected” and “unexpected” signals. When visual feedback is mismatched with motor command, the LC activity increases, representing an “unexpected” situation [75]. This phenomenon is consistent with the findings in mammalian studies that the LC is sensitive to the sensory feedback that deviates from expectation. Interestingly, the LC activation in zebrafish only encodes negative or “punishing” deviation when feedback is worse than expectation [75]. In contrast, in mammals, the LC NE neurons can encode reward prediction signals [133], which might be conveyed by projections from VTA to LC [51]. The VTA-LC pathway could have later evolved, but analogous circuits might exist in zebrafish. It would be informative to verify possible projections from DA neurons to the LC in zebrafish.

## 7. Conclusions

LC-NE, a vertebrate-specific neuromodulatory system, projects to the majority of brain areas. The broad LC projection forms a global network and regulates the neural activity locally. This local regulation targets both neurons and non-neuronal cells, such as astrocytes, and induces different functional changes depending on the difference of targeted cell types, brain regions, and animal classes. For example, astrocytes in the hippocampus of mammals, mediating NE induced place cell recruitment around the reward location [134,135,136], while astrocytes in the medulla oblongata of zebrafish, mediating NE induced regulation of the motor strategy [75]. The local specific modulation and its behavioral relevance can be highlighted and thoroughly addressed by utilizing different behavioral paradigms from different animals. Moreover, the widespread projections and local specific modulation of the LC-NE system can serve as a model for illustrating the organization principles of neuromodulation at the whole-brain scale.

**Figure 3:**
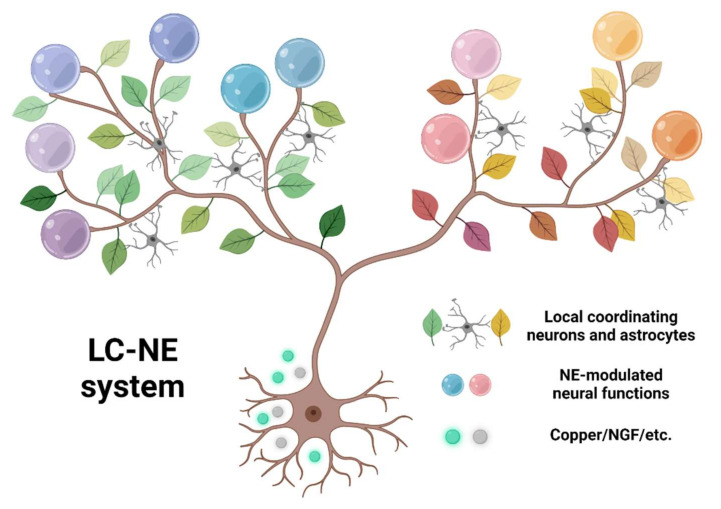
The conceptual framework of LC-NE system. The roots, LC specific gene expression, supporting varied activities and projections. The tree branches, extensive axonal projection of LC-NE system, broadcasting NE signal globally. The leaves, cooperating elements, such as local neurons or astrocytes. The fruits, the emergence of neural functions from localized neuromodulation. Created with BioRender.com (accessed on 8 January 2022).

We could imagine the LC-NE system as a tree (Figure 3). The widely projecting axons form branches broadcasting signals globally. Along each branch, the leaves are formed by local coordinating neural elements such as astrocytes, and the fruits of specific neural functions emerge from local coordinated NE neuromodulations. Under this framework, a whole-brain-wide survey from three aspects would be critical: (1) to characterize the organization principles of the “tree branches,” mesoscale single-cell reconstruction needs to be conducted. For example, brain regions could be functionally clustered, depending on if they are targeted by the same or different group of LC neurons. To deduce such principles from single reconstructed neurons, an oversampled number of cells would be necessary. Sparse labeling technique in zebrafish is widely used (200 LC neurons, 10 times the total LC neuron number) [137], and newly developed high-throughput imaging and reconstruction methods could potentially reconstruct 20,000 LC neurons in mice. (2) to decipher the communication rules between “branches” and “leaves,” simultaneously monitoring multiple neural components could outline the dynamics emerging from global to local interaction. The targets to be monitored include activity of LC soma and axon terminals, NE release from axons, and activity of local neurons and astrocytes. (3) to identify the “fruits” emerging from each branch, relevant behaviors need to be measured with simultaneous neural imaging, as mentioned in (2). Evidence accumulated from these three aspects will lead to a comprehensive description of the LC. Elucidation of this “tree” structure will provide a thorough understanding of LC and provide new insights regarding how a brain nucleus works with its global targets (Table 1).

## Figures and Tables

**Table 1:** The anatomical and functional features of the locus coeruleus in non-mammalian vertebrates.

Class	Contents	Species	References
Fish	Identified anatomical location, cell types, cell markers and cell morphology in the locus coeruleus (LC).	Electric fish, *Apteronotus leptorhynchus* Chondrostean fish, *Acipenser baeri* Chondrostean fish, *Huso huso* Short-lived fish, *Nothobranchius furzeri* Cartilaginous fish, *Hydrolagus colliei* Goldfish, *Carassius auratus* Zebrafish, *Danio rerio*	[8,17,58,62,63,78,82,86,138,139]
	Ascending and descending pathways.	Zebrafish, *Danio rerio*	[13,19,20,79,137]
	Circuit: hypocretin(Hcrt) and melanin-concentrating hormone(MCH) neurons project to the vicinity of the LC.	Goldfish, *Carassius auratus* Zebrafish, *Danio rerio*	[14,98,140]
	Regulating sleep and wakefulness.	Goldfish, *Carassius auratus* Zebrafish, *Danio rerio*	[14,93,98,140,141]
	Development: the LC cell appears between 8–12 hour post fertilization (hpf), differentiates between 24–48 hpf.	Zebrafish, *Danio rerio*	[7,77,78,79,81]
	Modulating brain states, including anxiety, anethesia, and passivity.	Zebrafish, *Danio rerio*	[15,75,99]
	Highly and specifically expression of copper transporter.	Zebrafish, *Danio rerio*	[93]
Amphibians	Ascending and descending pathways.	Newt, *Triturus cristatus* Newt, *Pleurodeles waltlii* Frog, *Xenopus laevis* Frog, *Rana perezi* Toad, *Bombian orientalis* Salamaner, *Necturus maculosus*	[21,27,142,143,144,145,146,147]
	Thermosensitivity and chemosensitivity.	Toad, *Bufo schneideri* Frog, *Lithobates catesbeianus*	[100,101,105,106]
	Controlling the melanotrope cells during background adaptation.	Frog, *Xenopus laevis*	[26,148]
	Expression of nitric oxide synthase.	Frog, *Xenopus laevis*	[149]
Reptiles	Ascending and descending pathways.	Turtle, *Pseudemys scripta elegans* Lizard, *Pogona vitticeps* Snake, *Python regius* Snake, *Python reticulatus* Green iguana, *Iguana iguana* Lizard, *Varanus exanthematicus* Lizard, *Gekko gecko*	[22,24,25,29,150,151,152,153,154,155]
	Reduced chemosensitivity at higher temperatures.	Lizard, *Varanus exanthematicus*	[107]
	Projections to claustrum: regulating slow-wave sleep.	Lizard, *Pogona vitticeps*	[153]
	Identified the anatomical location and measured the soma size.	Lizard, *Ctenosaura pectinata*	[156]
Birds	Ascending and descending pathways.	Pigeon, *Columba livia* Chicken, *Gallus gallus domesticus* Duck, *Anas platyrhinochos* L. Zebra finch, *Taeniopygia guttata*	[31,32,33,36,37,38,61,157,158,159]
	Regulating song quality, song preference and song variability.	Zebra finch, *Taeniopygia guttata*	[34,123,124,125,127,160,161,162,163,164,165,166,167,168,169]
	The LC activates during mobbing behavior.	Crow, *Corvus brachyrhynchos*	[170]
	Development: origin and migration.	Chicken, *Gallus gallus domesticus*	[5,171]
	High expression of neurotropin receptors; uptake neurotophin directly through axons and indirectly through tanycytes.	Chicken, *Gallus gallus domesticus*	[86,87]
	Modulation on body temperature and sleep	Pigeon, *Columba livia*	[172]

## Data Availability

All data obtained from published sources.

## References

[1] Smeets W.J.A.J., González A. (2000). Catecholamine systems in the brain of vertebrates: New perspectives through a comparative approach. Brain Res. Rev..

[2] Poe G.R., Foote S., Eschenko O., Johansen J.P., Bouret S., Aston-Jones G., Harley C.W., Manahan-Vaughan D., Weinshenker D., Valentino R. (2020). Locus coeruleus: A new look at the blue spot. Nat. Rev. Neurosci..

[3] Manger P.R., Eschenko O. (2021). The Mammalian Locus Coeruleus Complex—Consistencies and Variances in Nuclear Organization. Brain Sci..

[4] Barreiro-Iglesias A., Laramore C., Shifman M.I., Anadón R., Selzer M.E., Rodicio M.C. (2010). The sea lamprey tyrosine hydroxylase: cDNA cloning and in situ hybridization study in the brain. Neuroscience.

[5] Guglielmone R., Panzica G.C. (1982). Topographic, morphologic and developmental characterization of the nucleus loci coerulei in the chicken. Cell Tissue Res..

[6] Benarroch E.E. (2018). Locus coeruleus. Cell Tissue Res..

[7] Farrar M.J., Kolkman K.E., Fetcho J.R. (2018). Features of the structure, development, and activity of the zebrafish noradrenergic system explored in new CRISPR transgenic lines. J. Comp. Neurol..

[8] Holzschuh J., Ryu S., Aberger F., Driever W. (2001). Dopamine transporter expression distinguishes dopaminergic neurons from other catecholaminergic neurons in the developing zebrafish embryo. Mech. Dev..

[9] Castelino C.B., Schmidt M.F. (2010). What birdsong can teach us about the central noradrenergic system. J. Chem. Neuroanat..

[10] Sharma Y., Xu T., Graf W.M., Fobbs A., Sherwood C.C., Hof P.R., Allman J.M., Manaye K.F. (2009). Comparative anatomy of the locus coeruleus in humans and nonhuman primates. J. Comp. Neurol..

[11] Mouton P.R., Pakkenberg B., Gundersen H.J.G., Price D.L. (1994). Absolute number and size of pigmented locus coeruleus neurons in young and aged individuals. J. Chem. Neuroanat..

[12] Schwarz L.A., Luo L. (2015). Organization of the Locus Coeruleus-Norepinephrine System. Curr. Biol..

[13] Ma P.M. (1994). Catecholaminergic systems in the zebrafish. II. Projection pathways and pattern of termination of the locus coeruleus. J. Comp. Neurol..

[14] Prober D.A., Rihel J., Onah A.A., Sung R.-J., Schier A.F. (2006). Hypocretin/Orexin Overexpression Induces An Insomnia-Like Phenotype in Zebrafish. J. Neurosci..

[15] Lovett-Barron M., Andalman A.S., Allen W.E., Vesuna S., Kauvar I., Burns V.M., Deisseroth K. (2017). Ancestral Circuits for the Coordinated Modulation of Brain State. Cell.

[16] Jones B.E., Friedman L. (1983). Atlas of catecholamine perikarya, varicosities and pathways in the brainstem of the cat. J. Comp. Neurol..

[17] Ma P.M. (1994). Catecholaminergic systems in the zebrafish. I. Number, morphology, and histochemical characteristics of neurons in the locus coeruleus. J. Comp. Neurol..

[18] Northcutt R. (1981). Localization of neurons afferent to the telencephalon in a primitive bony fish, Polypterus palmas. Neurosci. Lett..

[19] Rink E., Wullimann M.F. (2004). Connections of the ventral telencephalon (subpallium) in the zebrafish (Danio rerio). Brain Res..

[20] Tay T.L., Ronneberger O., Ryu S., Nitschke R., Driever W. (2011). Comprehensive catecholaminergic projectome analysis reveals single-neuron integration of zebrafish ascending and descending dopaminergic systems. Nat. Commun..

[21] Dubé L., Clairambault P., Malacarne G. (1990). Striatal Afferents in the Newt Triturus cristatus. Brain, Behav. Evol..

[22] Wolters J., Donkelaar H.T., Verhofstad A. (1984). Distribution of catecholamines in the brain stem and spinal cord of the lizard Varanus exanthematicus: An immunohistochemical study based on the use of antibodies to tyrosine hydroxylase. Neuroscience.

[23] Sánchez-Camacho C., Peña J.J., González A. (2002). Catecholaminergic innervation of the septum in the frog: A combined immunohistochemical and tract-tracing study. J. Comp. Neurol..

[24] Bruce L.L., Butler A.B. (1984). Telencephalic connections in lizards. II. Projections to anterior dorsal ventricular ridge. J. Comp. Neurol..

[25] Hoogland P.V. (1982). Brainstem afferents to the thalamus in a lizard, Varanus exanthematicus. J. Comp. Neurol..

[26] Tuinhof R., Artero C., Fasolo A., Franzoni M., Donkelaar H.T., Wismans P., Roubos E. (1994). Involvement of retinohypothalamic input, suprachiasmatic nucleus, magnocellular nucleus and locus coeruleus in control of melanotrope cells of Xenopus Laevis: A retrograde and anterograde tracing study. Neuroscience.

[27] González A., Smeets W.J. (1993). Noradrenaline in the brain of the south african clawed frogXenopus laevis: A study with antibodies against noradrenaline and dopamine-β-hydroxylase. J. Comp. Neurol..

[28] Sánchez-Camacho C., Marín O., González A. (2002). Distribution and origin of the catecholaminergic innervation in the amphibian mesencephalic tectum. Vis. Neurosci..

[29] Bangma G.C., Donkelaar H.J.T. (1982). Afferent connections of the cerebellum in various types of reptiles. J. Comp. Neurol..

[30] Sánchez-Camacho C., Martín O., Ten Donkelaar H.J., González A. (2002). Descending supraspinal pathways in amphibians: III. Development of descending projections to the spinal cord in Xenopus laevis with emphasis on the catecholaminergic inputs. J. Comp. Neurol..

[31] Leutgeb S., Husband S., Riters L.V., Shimizu T., Bingman V.P. (1996). Telencephalic afferents to the caudolateral neostriatum of the pigeon. Brain Res..

[32] Kitt C.A., Brauth S.E. (1986). Telencephalic projections from midbrain and isthmal cell groups in the pigeon. I. Locus coeruleus and subcoeruleus. J. Comp. Neurol..

[33] Bagnoli P., Burkhalter A. (1983). Organization of the afferent projections to the Wulst in the pigeon. J. Comp. Neurol..

[34] Barr H.J., Wall E.M., Woolley S.C. (2021). Dopamine in the songbird auditory cortex shapes auditory preference. Curr. Biol..

[35] Dubé L., Parent A. (1981). The monoamine-containing neurons in avian brain: I. A study of the brain stem of the chicken (Gallus domesticus) by means of fluorescence and acetylcholinesterase histochemistry. J. Comp. Neurol..

[36] Rodman H.R., Karten H.J. (1995). Laminar distribution and sources of catecholaminergic input to the optic tectum of the pigeon (Columba livia). J. Comp. Neurol..

[37] Lucchi M.L., Callegari E., Barazzoni A.M., Chiocchetti R., Clavenzani P., Bortolami R. (1998). Cerebellar and spinal projections of the coeruleus complex in the duck: A fluorescent retrograde double-labeling study. Anat Rec..

[38] Mugnaini E., Dahl A.-L. (1975). Mode of distribution of aminergic fibers in the cerebellar cortex of the chicken. J. Comp. Neurol..

[39] Szabadi E. (2013). Functional neuroanatomy of the central noradrenergic system. J. Psychopharmacol..

[40] Rodenkirch C., Liu Y., Schriver B.J., Wang Q. (2018). Locus coeruleus activation enhances thalamic feature selectivity via norepinephrine regulation of intrathalamic circuit dynamics. Nat. Neurosci..

[41] Pickel V.M., Segal M., Bloom F.E. (1974). A radioautographic study of the efferent pathways of the nucleus locus coeruleus. J. Comp. Neurol..

[42] Uematsu A., Tan B.Z., Ycu E.A., Cuevas J.S., Koivumaa J., Junyent F., Kremer E., Witten I.B., Deisseroth K., Johansen J.P. (2017). Modular organization of the brainstem noradrenaline system coordinates opposing learning states. Nat. Neurosci..

[43] Takeuchi T., Duszkiewicz A.J., Sonneborn A., Spooner P.A., Yamasaki M., Watanabe M., Smith C.C., Fernández G., Deisseroth K., Greene R.W. (2016). Locus coeruleus and dopaminergic consolidation of everyday memory. Nature.

[44] Jones B.E., Yang T.-Z. (1985). The efferent projections from the reticular formation and the locus coeruleus studied by anterograde and retrograde axonal transport in the rat. J. Comp. Neurol..

[45] Clark F.M., Proudfit H.K. (1991). The projection of locus coeruleus neurons to the spinal cord in the rat determined by anterograde tracing combined with immunocytochemistry. Brain Res..

[46] Sluka K., Westlund K. (1992). Spinal projections of the locus coeruleus and the nucleus subcoeruleus in the Harlan and the Sasco Sprague-Dawley rat. Brain Res..

[47] Leong S.K., Shieh J.Y., Wong W.C. (1984). Localizing spinal-cord-projecting neurons in adult albino rats. J. Comp. Neurol..

[48] Berridge C.W., Waterhouse B.D. (2003). The locus coeruleus–noradrenergic system: Modulation of behavioral state and state-dependent cognitive processes. Brain Res. Rev..

[49] Bari B.A., Chokshi V., Schmidt K. (2020). Locus coeruleus-norepinephrine: Basic functions and insights into Parkinson’s disease. Neural. Regen Res..

[50] Waterhouse B.D., Navarra R.L. (2018). The locus coeruleus-norepinephrine system and sensory signal processing: A historical review and current perspectives. Brain Res..

[51] Ornstein K., Milon H., McRae-Degueurce A., Alvarez C., Berger B., Würzner H.P. (1987). Biochemical and radioautographic evidence for dopaminergic afferents of the locus coeruleus originating in the ventral tegmental area. J. Neural. Transm..

[52] Fritschy J.-M., Grzanna R. (1990). Demonstration of two separate descending noradrenergic pathways to the rat spinal cord: Evidence for an intragriseal trajectory of locus coeruleus axons in the superficial layers of the dorsal horn. J. Comp. Neurol..

[53] West W.L., Yeomans D.C., Proudfit H.K. (1993). The function of noradrenergic neurons in mediating antinociception induced by electrical stimulation of the locus coeruleus in two different sources of Sprague-Dawley rats. Brain Res..

[54] White S., Fung S., Barnes C. (1991). Norepinephrine effects on spinal motoneurons. Prog. Brain Res..

[55] Westlund K.N., Coulter J.D. (1980). Descending projections of the locus coeruleus and subcoeruleus/medial parabrachial nuclei in monkey: Axonal transport studies and dopamine-β-hydroxylase immunocytochemistry. Brain Res. Rev..

[56] Lewis D., Coote J. (1990). Excitation and inhibition of rat sympathetic preganglionic neurones by catecholamines. Brain Res..

[57] Shefchyk S.J. (2001). Sacral spinal interneurones and the control of urinary bladder and urethral striated sphincter muscle function. J. Physiol..

[58] Sas E., Maler L., Tinner B. (1990). Catecholaminergic systems in the brain of a gymnotiform teleost fish: An immunohistochemical study. J. Comp. Neurol..

[59] Kamei I., Shiosaka S., Senba E., Takagi H., Sakanaka M., Inagaki S., Takatsuki K., Nakai K., Imai H., Itakura T. (1981). Comparative anatomy of the distribution of catecholamines within the inferior olivary complex from teleosts to primates. J. Comp. Neurol..

[60] Echteler S.M., Saidel W.M. (1981). Forebrain Connections in the Goldfish Support Telencephalic Homologies with Land Vertebrates. Science.

[61] Castelino C.B., Diekamp B., Ball G.F. (2007). Noradrenergic projections to the song control nucleus area X of the medial striatum in male zebra finches (Taeniopygia guttata). J. Comp. Neurol..

[62] Hornby P.J., Piekut D.T. (1988). Immunoreactive dopamine β-hydroxylase in neuronal groups in the goldfish brain. Brain Behav. Evol..

[63] Adrio F., Anadón R., Rodríguez-Moldes I. (2002). Distribution of tyrosine hydroxylase (TH) and dopamine β-hydroxylase (DBH) immunoreactivity in the central nervous system of two chondrostean fishes (Acipenser baeri and Huso huso). J. Comp. Neurol..

[64] Moons L., D’Hondt E., Pijcke K., Vandesande F. (1995). Noradrenergic system in the chicken brain: Immunocytochemical study with antibodies to noradrenaline and dopamine-β-hydroxylase. J. Comp. Neurol..

[65] Totah N.K., Neves R.M., Panzeri S., Logothetis N.K., Eschenko O. (2018). The Locus Coeruleus Is a Complex and Differentiated Neuromodulatory System. Neuron.

[66] Chandler D.J., Gao W.-J., Waterhouse B.D. (2014). Heterogeneous organization of the locus coeruleus projections to prefrontal and motor cortices. Proc. Natl. Acad. Sci. USA.

[67] Chandler D.J., Jensen P., McCall J.G., Pickering A.E., Schwarz L.A., Totah N.K. (2019). Redefining Noradrenergic Neuromodulation of Behavior: Impacts of a Modular Locus Coeruleus Architecture. J. Neurosci..

[68] Kebschull J.M., da Silva P.G., Reid A.P., Peikon I.D., Albeanu D., Zador A.M. (2016). High-Throughput Mapping of Single-Neuron Projections by Sequencing of Barcoded RNA. Neuron.

[69] Winnubst J., Bas E., Ferreira T.A., Wu Z., Economo M.N., Edson P., Arthur B.J., Bruns C., Rokicki K., Schauder D. (2019). Reconstruction of 1,000 Projection Neurons Reveals New Cell Types and Organization of Long-Range Connectivity in the Mouse Brain. Cell.

[70] Kunst M., Laurell E., Mokayes N., Kramer A., Kubo F., Fernandes A.M., Förster D., Maschio M.D., Baier H. (2019). A Cellular-Resolution Atlas of the Larval Zebrafish Brain. Neuron.

[71] Randlett O., Wee C., Naumann E.A., Nnaemeka O., Schoppik D., Fitzgerald J.E., Portugues R., Lacoste A., Riegler C., Engert F. (2015). Whole-brain activity mapping onto a zebrafish brain atlas. Nat. Methods.

[72] Tabor K.M., Marquart G.D., Hurt C., Smith T.S., Geoca A.K., Bhandiwad A.A., Subedi A., Sinclair J.L., Rose H.M., Polys N.F. (2019). Brain-wide cellular resolution imaging of Cre transgenic zebrafish lines for functional circuit-mapping. eLife.

[73] Ahrens M.B., Orger M.B., Robson D.N., Li J.M., Keller P.J. (2013). Whole-brain functional imaging at cellular resolution using light-sheet microscopy. Nat. Methods.

[74] Dunn T.W., Mu Y., Narayan S., Randlett O., Naumann E.A., Yang C.-T., Schier A.F., Freeman J., Engert F., Ahrens M.B. (2016). Brain-wide mapping of neural activity controlling zebrafish exploratory locomotion. eLife.

[75] Mu Y., Bennett D., Rubinov M., Narayan S., Yang C.-T., Tanimoto M., Mensh B.D., Looger L.L., Ahrens M.B. (2019). Glia Accumulate Evidence that Actions Are Futile and Suppress Unsuccessful Behavior. Cell.

[76] Feng J., Zhang C., Lischinsky J.E., Jing M., Zhou J., Wang H., Zhang Y., Dong A., Wu Z., Wu H. (2019). A Genetically Encoded Fluorescent Sensor for Rapid and Specific In Vivo Detection of Norepinephrine. Neuron.

[77] Du Y., Guo Q., Shan M., Wu Y., Huang S., Zhao H., Hong H., Yang M., Yang X., Ren L. (2016). Spatial and Temporal Distribution of Dopaminergic Neurons during Development in Zebrafish. Front. Neuroanat..

[78] McLean D.L., Fetcho J.R. (2004). Ontogeny and innervation patterns of dopaminergic, noradrenergic, and serotonergic neurons in larval zebrafish. J. Comp. Neurol..

[79] Schweitzer J., Löhr H., Filippi A., Driever W. (2012). Dopaminergic and noradrenergic circuit development in zebrafish. Dev. Neurobiol..

[80] Becker C.G., Becker T. (2007). Model Organisms in Spinal Cord Regeneration.

[81] Rink E., Wullimann M.F. (2002). Development of the catecholaminergic system in the early zebrafish brain: An immunohistochemical study. Dev. Brain Res..

[82] Sun P., Wang J., Zhang M., Duan X., Wei Y., Xu F., Ma Y., Zhang Y.-H. (2020). Sex-Related Differential Whole-Brain Input Atlas of Locus Coeruleus Noradrenaline Neurons. Front. Neural Circuits.

[83] Mulvey B., Bhatti D.L., Gyawali S., Lake A.M., Kriaucionis S., Ford C.P., Bruchas M.R., Heintz N., Dougherty J.D. (2018). Neurons in the Mouse Locus Coeruleus. Cell Rep..

[84] Guo S., Brush J., Teraoka H., Goddard A., Wilson S.W., Mullins M.C., Rosenthal A. (1999). Development of noradrenergic neurons in the zebrafish hindbrain requires BMP, FGF8, and the homeodomain protein soulless/Phox2a. Neuron.

[85] Stuesse S.L., Cruce W.L.R. (1991). Immunohistochemical localization of serotoninergic, enkephalinergic, and catecholaminergic cells in the brainstem and diencephalon of a cartilaginous fish, hydrolagus colliei. J. Comp. Neurol..

[86] Von Bartheld C., Schober A., Kinoshita Y., Williams R., Ebendal T., Bothwell M. (1995). Noradrenergic neurons in the locus coeruleus of birds express TrkA, transport NGF, and respond to NGF. J. Neurosci..

[87] Feng C.-Y., Wiggins L.M., von Bartheld C.S. (2011). The Locus Ceruleus Responds to Signaling Molecules Obtained from the CSF by Transfer through Tanycytes. J. Neurosci..

[88] Schwab M., Otten U., Agid Y., Thoenen H. (1979). Nerve growth factor (NGF) in the rat CNS: Absence of specific retrograde axonal transport and tyrosine hydroxylase induction in locus coeruleus and substantia nigra. Brain Res..

[89] Warren P.J., Earl C.J., Thompson R.H.S. (1960). The distribution of copper in human brain. Brain.

[90] Vendelboe T.V., Harris P., Zhao Y., Walter T.S., Harlos K., El Omari K., Christensen H.E. (2016). The crystal structure of human dopamine β-hydroxylase at 2.9 Å resolution. Sci. Adv..

[91] Kaler S.G. (2011). ATP7A-related copper transport diseases—emerging concepts and future trends. Nat. Rev. Neurol..

[92] Madsen E., Gitlin J.D. (2007). Copper and Iron Disorders of the Brain. Annu. Rev. Neurosci..

[93] Xiao T., Ackerman C.M., Carroll E.C., Jia S., Hoagland A., Chan J., Thai B., Liu C.S., Isacoff E., Chang C.J. (2018). Copper regulates rest-activity cycles through the locus coeruleus-norepinephrine system. Nat. Chem. Biol..

[94] Schmidt K., Ralle M., Schaffer T., Jayakanthan S., Bari B., Muchenditsi A., Lutsenko S. (2018). ATP7A and ATP7B copper transporters have distinct functions in the regulation of neuronal dopamine-β-hydroxylase. J. Biol. Chem..

[95] Davies K.M., Bohic S., Carmona A., Ortega R., Cottam V., Hare D.J., Finberg J.P., Reyes S., Halliday G.M., Mercer J.F. (2014). Copper pathology in vulnerable brain regions in Parkinson’s disease. Neurobiol. Aging.

[96] Benhamla T., Tirouche Y., Abaoub-Germain A., Theodore F. (2007). Mode d’entrée psychiatrique dans la maladie de Wilson: À propos d’un cas à début tardif. L’encéphale.

[97] Montes S., Rivera-Mancía S., Díaz-Ruíz A., Tristán-López L., Ríos C. (2014). Copper and Copper Proteins in Parkinson’s Disease. Oxidative Med. Cell. Longev..

[98] Singh C., Oikonomou G., Prober D.A. (2015). Norepinephrine is required to promote wakefulness and for hypocretin-induced arousal in zebrafish. eLife.

[99] Du W.-J., Zhang R., Li J., Zhang B.-B., Peng X.-L., Cao S., Yuan J., Yuan C.-D., Yu T., Du J.-L. (2018). The Locus Coeruleus Modulates Intravenous General Anesthesia of Zebrafish via a Cooperative Mechanism. Cell Rep..

[100] Santin J.M., Watters K.C., Putnam R.W., Hartzler L.K. (2013). Temperature influences neuronal activity and CO2/pH sensitivity of locus coeruleus neurons in the bullfrog, Lithobates catesbeianus. Am. J. Physiol. Integr. Comp. Physiol..

[101] Santin J.M., Hartzler L.K. (2015). Activation state of the hyperpolarization-activated current modulates temperature-sensitivity of firing in locus coeruleus neurons from bullfrogs. Am. J. Physiol. Integr. Comp. Physiol..

[102] Kobayashi S., Takahashi T. (1993). Whole-cell properties of temperature-sensitive neurons in rat hypothalamic slices. Proc. R. Soc. B Boil. Sci..

[103] Keenan C.L., Chu N.-S. (1987). Thermosensitivity of dorsal raphe neurons in vitro. Brain Res..

[104] Morilak D.A., Fornal C.A., Jacobs B.L. (1987). Effects of physiological manipulations on locus coeruleus neuronal activity in freely moving cats. II. Cardiovascular challenge. Brain Res..

[105] Noronha-De-Souza C.R., Bícego K.C., Michel G., Glass M.L., Branco L.G.S., Gargaglioni L.H. (2006). Locus coeruleus is a central chemoreceptive site in toads. Am. J. Physiol. Integr. Comp. Physiol..

[106] Gargaglioni L.H., Hartzler L.K., Putnam R.W. (2010). The locus coeruleus and central chemosensitivity. Respir. Physiol. Neurobiol..

[107] Zena L.A., Fonseca E.M., Santin J.M., Porto L., Gargaglioni L., Bícego K.C., Hartzler L.K. (2016). Effect of temperature on chemosensitive locus coeruleus neurons of Savannah monitor lizards Varanus exanthematicus. J. Exp. Biol..

[108] Filosa J.A., Dean J.B., Putnam R.W. (2002). Role of intracellular and extracellular pH in the chemosensitive response of rat locus coeruleus neurones. J. Physiol..

[109] Oyamada Y., Ballantyne D., Mückenhoff K., Scheid P. (1998). Respiration-modulated membrane potential and chemosensitivity of locus coeruleus neurones in thein vitrobrainstem-spinal cord of the neonatal rat. J. Physiol..

[110] Gargaglioni L.H., Meier J.T., Branco L.G.S., Milsom W.K. (2007). Role of midbrain in the control of breathing in anuran amphibians. Am. J. Physiol. Integr. Comp. Physiol..

[111] Mitchell C. (1989). Respiratory failure!. Med. J. Aust..

[112] Gallup A.C., Gallup G.G. (2007). Yawning as a Brain Cooling Mechanism: Nasal Breathing and Forehead Cooling Diminish the Incidence of Contagious Yawning. Evol. Psychol..

[113] Gallup A.C., Eldakar O.T. (2013). The thermoregulatory theory of yawning: What we know from over 5 years of research. Front. Neurosci..

[114] Da Silva G.S.F., Glass M.L., Branco L.G. (2013). Temperature and respiratory function in ectothermic vertebrates. J. Therm. Biol..

[115] Schepers R.J., Ringkamp M. (2010). Thermoreceptors and thermosensitive afferents. Neurosci. Biobehav. Rev..

[116] Rosenthal T. (1948). The effect of temperature on the pH of blood and plasma in vitro. J. Biol. Chem..

[117] Zwart A., Kwant G., Oeseburg B., Zijlstra W.G. (1984). Human whole-blood oxygen affinity: Effect of temperature. J. Appl. Physiol..

[118] Schiff S.J., Somjen G.G. (1985). The effects of temperature on synaptic transmission in hippocampal tissue slices. Brain Res..

[119] Howell B.J., Baumgardner F.W., Bondi K., Rahn H. (1970). Acid-base balance in cold-blooded vertebrates as a function of body temperature. Am. J. Physiol. Content.

[120] Whitford W.G. (1973). The Effects of Temperature on Respiration in the Amphibia. Am. Zool..

[121] Biancardi V., Bícego K.C., Almeida M.C., Gargaglioni L.H. (2007). Locus coeruleus noradrenergic neurons and CO2 drive to breathing. Pflug. Arch. Eur. J. Phy..

[122] Sleiman F., Saab S. (1995). Influence of environment on respiration, heart rate and body temperature of filial crosses compared to local Awassi sheep. Small Rumin. Res..

[123] Ikeda M.Z., Jeon S.D., Cowell R.A., Remage-Healey L. (2015). Norepinephrine Modulates Coding of Complex Vocalizations in the Songbird Auditory Cortex Independent of Local Neuroestrogen Synthesis. J. Neurosci..

[124] Poirier C., Boumans T., Vellema M., de Groof G., Charlier T.D., Verhoye M., van der Linden A., Balthazart J. (2011). Own Song Selectivity in the Songbird Auditory Pathway: Suppression by Norepinephrine. PLoS ONE.

[125] Sheldon Z.P., Castelino C.B., Glaze C.M., Bibu S.P., Yau E., Schmidt M.F. (2020). Regulation of vocal precision by noradrenergic modulation of a motor nucleus. J. Neurophysiol..

[126] Farries M.A. (2004). The Avian Song System in Comparative Perspective. Ann. N. Y. Acad. Sci..

[127] Alvarado J.S., Goffinet J., Michael V., Liberti W., Hatfield J., Gardner T., Pearson J., Mooney R. (2021). Neural dynamics underlying birdsong practice and performance. Nature.

[128] Sizemore M., Perkel D.J. (2008). Noradrenergic and GABAB Receptor Activation Differentially Modulate Inputs to the Premotor Nucleus RA in Zebra Finches. J. Neurophysiol..

[129] Vazey E.M., Moorman D.E., Aston-Jones G. (2018). Phasic locus coeruleus activity regulates cortical encoding of salience information. Proc. Natl. Acad. Sci. USA.

[130] Duzel E., Guitart-Masip M. (2013). Not So Uncertain at Last: Locus Coeruleus and Decision Making. Neuron.

[131] Yu A.J., Dayan P. (2005). Uncertainty, Neuromodulation, and Attention. Neuron.

[132] Payzan-LeNestour E., Dunne S., Bossaerts P., O’Doherty J.P. (2013). The Neural Representation of Unexpected Uncertainty during Value-Based Decision Making. Neuron.

[133] Bouret S., Richmond B.J. (2015). Sensitivity of Locus Ceruleus Neurons to Reward Value for Goal-Directed Actions. J. Neurosci..

[134] Kaufman A.M., Geiller T., Losonczy A. (2020). A Role for the Locus Coeruleus in Hippocampal CA1 Place Cell Reorganization during Spatial Reward Learning. Neuron.

[135] Paukert M., Agarwal A., Cha J., Doze V.A., Kang J.U., Bergles D.E. (2014). Norepinephrine Controls Astroglial Responsiveness to Local Circuit Activity. Neuron.

[136] Deemyad T., Lüthi J., Spruston N. (2018). Astrocytes integrate and drive action potential firing in inhibitory subnetworks. Nat. Commun..

[137] Helmbrecht T.O., Maschio M.D., Donovan J.C., Koutsouli S., Baier H. (2018). Topography of a Visuomotor Transformation. Neuron.

[138] Borgonovo J., Ahumada-Galleguillos P., Oñate-Ponce A., Allende-Castro C., Henny P., Concha M.L. (2021). Organization of the Catecholaminergic System in the Short-Lived Fish Nothobranchius furzeri. Front. Neuroanat..

[139] Ma P.M., Lopez M. (2003). Consistency in the number of dopaminergic paraventricular organ-accompanying neurons in the posterior tuberculum of the zebrafish brain. Brain Res..

[140] Huesa G., Pol A.N.V.D., Finger T.E. (2005). Differential distribution of hypocretin (orexin) and melanin-concentrating hormone in the goldfish brain. J. Comp. Neurol..

[141] Singh C., Rihel J., Prober D.A. (2017). Neuropeptide Y Regulates Sleep by Modulating Noradrenergic Signaling. Curr. Biol..

[142] Sánchez-Camacho C., Marín O., López J., Moreno N., Smeets W., Donkelaar H.T., González A. (2002). Origin and development of descending catecholaminergic pathways to the spinal cord in amphibians. Brain Res. Bull..

[143] Marín O., González A., Smeets W.J.A.J. (1997). Basal ganglia organization in amphibians: Efferent connections of the striatum and the nucleus accumbens. J. Comp. Neurol..

[144] González A., Smeets W.J.A.J. (1991). Comparative analysis of dopamine and tyrosine hydroxylase immunoreactivities in the brain of two amphibians, the anuranRana ridibunda and the urodelePleurodeles waltlii. J. Comp. Neurol..

[145] Freudenmacher L., von Twickel A., Walkowiak W. (2019). The habenula as an evolutionary conserved link between basal ganglia, limbic, and sensory systems—A phylogenetic comparison based on anuran amphibians. J. Comp. Neurol..

[146] Dubé L., Parent A. (1982). The organization of monoamine-containing neurons in the brain of the salamander, Necturus maculosus. J. Comp. Neurol..

[147] Marin O., Smeets W.J., González A. (1996). Do amphibians have a true locus coeruleus?. Neuroreport.

[148] Kolk S.M., Berghs C.A.F.M., Vaudry H., Verhage M., Roubos E.W. (2001). Physiological Control of Xunc18 Expression in Neuroendocrine Melanotrope Cells of Xenopus laevis. Endocrinology.

[149] Brüning G., Mayer B. (1996). Localization of nitric oxide synthase in the brain of the frog, Xenopus laevis. Brain Res..

[150] Donkelaar H.J., Huizen R. (1988). Brain stem afferents to the anterior dorsal ventricular ridge in a lizard (Varanus exanthematicus). Anat. Embryol..

[151] Ouimet C.C., Patrick R.L., Ebner F.F. (1985). The projection of three extrathalamic cell groups to the cerebral cortex of the turtlePseudemys. J. Comp. Neurol..

[152] Siemen M., Künzle H. (1994). Connections of the basal telencephalic areas c and d in the turtle brain. Anat. Embryol..

[153] Norimoto H., Fenk L., Li H.-H., Tosches M.A., Gallego-Flores T., Hain D., Reiter S., Kobayashi R., Macias A., Arends A. (2020). A claustrum in reptiles and its role in slow-wave sleep. Nature.

[154] Donkelaar H.J., Bangma G.C., Huizen R.B.-V. (1983). Reticulospinal and vestibulospinal pathways in the snake Python regius. Anat. Embryol..

[155] Welker E., Hoogland P.V., Lohman A.H.M. (1983). Tectal connections inPython reticulatus. J. Comp. Neurol..

[156] Ayala-Guerrero F., Mexicano G. (2007). Topographical distribution of the locus coeruleus and raphe nuclei in the lizard Ctenosaura pectinata: Functional implications on sleep. Comp. Biochem. Physiol. Part A Mol. Integr. Physiol..

[157] Kitt C.A., Brauth S.E. (1986). Telencephalic projections from midbrain and isthmal cell groups in the pigeon. II. The nigral complex. J. Comp. Neurol..

[158] Atoji Y., Saito S., Wild J.M. (2006). Fiber connections of the compact division of the posterior pallial amygdala and lateral part of the bed nucleus of the stria terminalis in the pigeon (Columba livia). J. Comp. Neurol..

[159] Parent A. (1984). Functional Anatomy and Evolution of Monoaminergic Systems. Am. Zool..

[160] Ribeiro S., Mello C. (2000). Gene Expression and Synaptic Plasticity in the Auditory Forebrain of Songbirds. Learn. Mem..

[161] Wade J., Lampen J., Qi L., Tang Y.P. (2012). Norepinephrine inhibition in juvenile male zebra finches modulates adult song quality. Brain Res. Bull..

[162] Matragrano L.L., LeBlanc M.M., Chitrapu A., Blanton Z.E., Maney D.L. (2013). Testosterone alters genomic responses to song and monoaminergic innervation of auditory areas in a seasonally breeding songbird. Dev. Neurobiol..

[163] Soha J.A., Shimizu T., Doupe A.J. (1996). Development of the catecholaminergic innervation of the song system of the male zebra finch. J. Neurobiol..

[164] Waterman S.A., Harding C.F. (2008). Neurotoxic effects of DSP-4 on the central noradrenergic system in male zebra finches. Behav. Brain Res..

[165] Barr H.J., Woolley S.C. (2018). Developmental auditory exposure shapes responses of catecholaminergic neurons to socially-modulated song. Sci. Rep..

[166] Velho T.A.F., Lü K., Ribeiro S., Pinaud R., Vicario D., Mello C.V. (2012). Noradrenergic Control of Gene Expression and Long-Term Neuronal Adaptation Evoked by Learned Vocalizations in Songbirds. PLoS ONE.

[167] Lynch K.S., Diekamp B., Ball G.F. (2008). Catecholaminergic cell groups and vocal communication in male songbirds. Physiol. Behav..

[168] Barclay S.R., Harding C.F., Waterman S.A. (1992). Correlations between catecholamine levels and sexual behavior in male zebra finches. Pharmacol. Biochem. Behav..

[169] Lynch K.S., Diekamp B., Ball G.F. (2012). Colocalization of Immediate Early Genes in Catecholamine Cells after Song Exposure in Female Zebra Finches (Taeniopygia guttata). Brain, Behav. Evol..

[170] Cross D., Marzluff J.M., Palmquist I., Minoshima S., Shimizu T., Miyaoka R. (2013). Distinct neural circuits underlie assessment of a diversity of natural dangers by American crows. Proc. R. Soc. B Boil. Sci..

[171] Aroca P., Lorente-Cánovas B., Mateos F.R., Puelles L. (2006). Locus coeruleus neurons originate in alar rhombomere 1 and migrate into the basal plate: Studies in chick and mouse embryos. J. Comp. Neurol..

[172] Yekimova I.V., Pastukhov I.F. (2002). The GABAergic midbrain system is involved in the control of sleep and temperature homeostasis in pigeons. Dokl. Biol. Sci..

